# Clinical application of ^18^F-FCH PET/CT in the diagnosis and treatment of hyperparathyroidism

**DOI:** 10.3389/fendo.2023.1100056

**Published:** 2023-04-11

**Authors:** Shuang Liu, Mengdan Li, Hua Pang, Rui Zuo, Lingquan Kong, Zhengjie Wang, Wenbo Li, Zhu Xia, Dong Wang, Lu Xu

**Affiliations:** ^1^ Department of Nuclear Medicine, The First Affiliated Hospital of Chongqing Medical University, Chongqing, China; ^2^ Department of Breast Thyroid Surgery, The First Affiliated Hospital of Chongqing Medical University, Chongqing, China; ^3^ Department of Ultrasound Medicine, The First Affiliated Hospital of Chongqing Medical University, Chongqing, China

**Keywords:** hyperparathyroidism, ^18^F-FCH PET/CT, quantitative study, diagnosis, imaging time selection

## Abstract

**Objective:**

We evaluated the difference in parathyroid visualization on ^18^F-FCH PET/CT images obtained at 5 and 60 min, and quantitatively analyzed the mode of FCH uptake at different time points, to determine the best imaging time for FCH PET/CT.

**Methods:**

This retrospective study included 73 patients with hyperparathyroidism (HPT) who underwent ^18^F-FCH PET/CT imaging between December 2017 and December 2021. The diagnostic efficiency of 5- and 60-min dual time point imaging for the diagnosis of hyperparathyroidism and parathyroid adenoma and hyperplasia, were compared using visual and quantitative analyses.

**Results:**

Dual-time ^18^F-FCH PET/CT imaging visual analysis had diagnostic value for HPT. The receiver operating characteristic curve of PET/CT quantitative parameters for the diagnosis of HPT and lesions showed that the parathyroid/thyroid SUVmax ratio for 60-min imaging had a higher sensitivity and specificity (based on patient, sensitivity: 90.90% and specificity: 85.71%; based on focus, sensitivity: 83.06% and specificity: 85.71%) compared to that for 5-min imaging. PET/CT quantitative parameters can distinguish parathyroid adenoma and hyperplasia. The 60-min parathyroid SUVmax value had the highest diagnostic value (cutoff: 3.945; area under the curve: 0.783).

**Conclusion:**

The quantitative parameters of 60min ^18^F-FCH PET/CT have more advantages in aiding in the pathologica diagnosis and clinical treatment of HPT.

## 1 Introduction

Hyperparathyroidism (HPT) is a complex disease characterized by abnormal calcium and phosphorus metabolism due to excessive parathyroid hormone (PTH) secretion. Early stages of the disease are not associated with any symptoms (1). In HPT, increased blood and urine calcium levels, and abnormal calcium and phosphorus metabolism may lead to renal calcinosis, urolithiasis, osteopathy, neuropsychiatric diseases, gastrointestinal disorders, weakness, spasms, muscle pain, and other neuromuscular manifestations. It is diagnosed on the basis of serum calcium and PTH levels. Primary HPT (pHPT) is primarily treated by surgical resection of the parathyroid glands. Secondary and tertiary HPT (sHPT and tHPT, respectively) usually require preoperative localization of the parathyroid glands to remove most of the hyperplastic parathyroid tissue (2, 3). Several surgical methods are available that vary in terms of the operation time, postoperative pain, and risk of postoperative symptomatic hypocalcemia (4–6); therefore, accurately determining the preoperative location of the parathyroid glands is essential for a successful operation. Parathyroid ultrasound and ^99m^Tc-methoxyisobutyl isonitrile (^99m^Tc-MIBI) imaging are the first-line diagnostic tests for preoperative localization. However, the accuracy of these investigations is affected by the imaging technique, operator’s experience, size of the focus, and the number of lesions (i.e., single or multiple lesions). Almost 10–30% of the diagnoses are missed with these investigations (7, 8). ^18^F-fluorocholine (^18^F-FCH) is a phospholipid analog that integrates a radiolabeled choline molecule into the newly synthesized proliferative cell membrane, leading to increased uptake of choline into the hyperparathyroid tissue due to upregulation of choline kinase (9). An increasing number of studies have found that ^18^F-FCH PET/CT imaging is useful for preoperative localization of the parathyroid glands; compared to ^99m^Tc-MIBI and ultrasound, ^18^F-FCH PET/CT is associated with a lower radiation dose, shorter imaging time, and higher spatial resolution and sensitivity (10–12). Additionally, ^18^F-FCH PET/CT can be used systematically for the preliminary evaluation of HPT in patients with suspected pHPT (13, 14). Its use has also been recently described for tHPT and sHPT (2).

According to a recent meta-study (15), a total of thousands of patients underwent ^18^F-FCH PET/CT, which is more effective than first-line examinations such as ultrasound and ^99m^Tc-MIBI imaging in many studies, and the guidelines recommend ^18^F-FCH PET/CT as another first-line imaging method (16). Previous studies (17) performed FCH PET/CT imaging at different time points after injection (0–120 min) and found inconclusive results regarding the optimal imaging time. Most studies have compared images obtained at 5 and 60 min (18, 19), and have reported conflicting results. Few studies have found that ^18^F-FCH PET/CT has more advantages in the diagnosis of parathyroid adenomas, but its usefulness in diagnosis of hyperplasia and differentiating parathyroid adenoma from hyperplasia has not been established. We visually compared the parathyroid glands in HPT patients on dual-time FCH PET/CT images obtained at 5 and 60 min, quantitatively analyzed the FCH uptake patterns at different times, and compared the parameters between parathyroid adenoma and hyperplasia to determine the optimal imaging time of FCH PET/CT, improve the diagnostic rates of parathyroid adenoma and hyperplasia, and provide support for its usefulness for diagnosis and the treatment of HPT patients.

## 2 Materials and methods

### 2.1 Study population

In total, 135 patients who underwent FCH PET/CT between December 2017 and December 2021 were evaluated for inclusion in the study. In accordance with the study eligibility criteria, 73 patients were enrolled (33 males and 40 females; mean age: 51.00 ± 14.10 years). The study included patients who underwent parathyroid surgery and had complete pathological data for 5- and 60-min FCH PET/CT. Patients without parathyroid surgery, or no pathology report, and those who underwent only one ^18^F-FCH PET/CT imaging session, were excluded. Postoperative pathological analyses of the 138 tissues showed adenoma, hyperplasia, parathyroid tissue, and thyroid papillary carcinoma in 23 (16.67%), 100 (72.46%), 7 (5.07%), and 8 (5.80%) samples, respectively.

### 2.2 ^18^F-FCH PET/CT imaging

PET/CT imaging was performed using a Gemini TF64 PET/CT Scanner (Philips Healthcare, Best, the Netherlands). Images of the neck and upper mediastinum were obtained at 5 and 60 min after the intravenous injection of 5 mCi of 18F-FCH using an accelerator (radiochemical purity > 95%; Sumitomo Corp., Kagawa, Japan). PET was performed in two recumbent positions (5 min/supine position). A low-dose CT image was obtained using the standard parameters of 100 mA, 120 Kv, 512 × 512 matrix, and 2-mm layer thickness. The maximum intensity projection and fusion images were obtained using computer iterative reconstruction and attenuation correction.

The FCH PET/CT images obtained at different time points were analyzed by two experienced nuclear medicine specialists with > 8 years of experience in parathyroid imaging. Disagreements were resolved by discussion. The location of the lesions reported by pathological analysis was compared to the PET/CT images. In previous studies (20–22), thyroid and neck muscle are the main references for comparison of parathyroid metabolism. Considering that muscle tissue is more prone to change due to activities before the examination, the thyroid is used as a reference to reflect parathyroid metabolism. In visual analysis, uptake by the parathyroid glands that was absent or significantly lower than that by the thyroid bed was considered no uptake and was considered negative. Uptake by the parathyroid glands that was slightly higher than or equivalent to that of the thyroid bed was considered suspicious uptake, uptake by the parathyroid glands that was significantly higher than that by the thyroid bed was considered obvious uptake, both of which were considered positive.

According to the postoperative pathological reports, two nuclear medicine physicians located the parathyroid gland on PET and CT images one by one, drew the corresponding parathyroid gland, and placed the sketched shape in the adjacent normal thyroid gland manually in the software to get the SUVmax of parathyroid gland and thyroid. The maximum standard uptake values (parathyroid SUVmax [P SUVmax] and thyroid SUVmax [T SUVmax]) of the parathyroid lesions and thyroid gland at 5 and 60 min were quantified. Additionally, the parathyroid/thyroid (P/T) SUVmax was calculated. The most prominent lesions were used to determine the character of the patient for patient-based analysis. If there are multiple lesions in the same patient, the most obvious lesions are determined by the visual assessment of two observers in the visual analysis; in quantitative analysis, the lesions with the largest SUVmax were regarded as the most obvious lesions. The imaging findings were compared to those of the postoperative pathological examination to determine the diagnostic efficiency of 5- and 60-min imaging.

With intraoperative localization and postoperative histology as reference standards, imaging results were interpreted as follows: true-positive, location with regional tracer uptake as well as pathological confirmation of parathyroid hyperplasia and/or adenoma; false-positive, location with regional tracer uptake, and histologic findings other than parathyroid hyperplasia and adenoma; true-negative, location without regional tracer uptake, histologic findings other than parathyroid hyperplasia and adenoma; false-negative, location without regional tracer uptake, but pathologically confirmed as parathyroid hyperplasia and/or adenoma.

### 2.3 Statistical analysis

Data analyses were performed using SPSS software (version 26.0; IBM Corp., Armonk, NY, USA) and GraphPad Prism 9.0.0(GraphPad Software, San Diego, California). The agreement between observers was tested using the Kappa test (Kappa values < 0: inconsistent, 0.0–0.20: slightly consistent, 0.21–0.40: moderately consistent, 0.41–0.60: moderately consistent, 0.61–0.80: basically consistent, and 0.81–1.00: almost identical).

The groups were compared using the paired *t*-test, χ^2^ test, or Mann-Whitney U test, as appropriate. The paired *t*-test was used to compare the parameters on 5- and 60-min imaging. A receiver operating characteristic (ROC) curve was constructed to determine the optimal cutoff value and diagnostic efficiency of the imaging methods. The McNemar test was used to compare the effectiveness of the visual analysis.

## 3 Results

### 3.1 General characteristics

The patients were divided into pHPT (42 cases) and sHPT (31 cases) groups according to their clinical history and examination. There were significant differences between the groups in terms of the presence of osteoporosis and PTH, bone-specific alkaline phosphatase, serum calcium, serum phosphorus, and 25-OH vitamin D levels (*p* = 0.001, = 0.000, = 0.000, = 0.004, = 0.000, and = 0.009, respectively). However, there was no significant difference in the P SUVmax, T SUVmax, and P/T SUVmax on 5- and 60-min images between the two groups (**Table 1**).

**Table 1:** General clinicopathological features of the patients.

	Patients (N=73)	pHPT (N=43, 58.90%)	sHPT (N=30, 41.09%)	*t*/*χ* ^2^	*p*
	51.00 ± 14.10	51.02 ± 14.78	50.97 ± 13.37	0.302	0.764
Gender					
M	33 (45.21%)	20	13	0.233	0.630
F	40 (54.79%)	23	17		
Hypercalcemia					
Yes	52 (71.24%)	31	21	0.038	0.846
No	21 (28.76%)	12	9		
Osteoporosis					
Yes	42 (57.53%)	18	24	10.521	0.001^*^
No	31 (42.47%)	25	6		
Pathology					
Adenoma	21 (61.64%)	18	3	9.028	0.011^*^
Hyperplastic	45 (28.77%)	21	24		
Others	7 (9.59%)	4	3		
PTH	822.77 ± 955.18	388.33 ± 518.29	1445.46 ± 1093.24	-5.524	0.000^*^
CT	6.68 ± 18.62	3.45 ± 6.21	11.29 ± 27.70	-1.799	0.076
BAP	50.28 ± 43.14	32.71 ± 31.54	75.45 ± 45.46	-4.747	0.000^*^
Ca	2.73 ± 0.44	2.85 ± 0.50	2.56 ± 0.27	2.944	0.004^*^
P	1.18 ± 0.53	0.96 ± 0.30	1.49 ± 0.62	-4.868	0.000^*^
25-OH VD	17.68 ± 11.27	14.86 ± 5.77	21.87 ± 15.55	-2.7	0.009^*^
5min P SUVmax	4.65 ± 2.09	4.37 ± 1.92	5.06 ± 2.29	-1.412	0.162
5min T SUVmax	1.83 ± 0.72	1.85 ± 0.62	1.79 ± 0.85	0.335	0.738
5min P/T	2.76 ± 1.41	2.53 ± 1.42	3.09 ± 1.34	-1.685	0.096
60min P SUVmax	4.23 ± 1.83	3.99 ± 1.78	4.56 ± 1.88	-1.306	0.196
60min T SUVmax	1.65 ± 0.76	1.68 ± 0.73	1.60 ± 0.74	0.549	0.585
60min P/T	2.79 ± 1.41	2.56 ± 1.43	3.10 ± 1.34	-1.601	0.114

* P < 0.05, with statisticalsignificance.

### 3.2 Visual analysis of the ^18^F-FCH PET/CT images

Our study compared the sensitivity, specificity, positive predictive value, negative predictive value, and accuracy of the visual analysis of 5- and 60-min FCH PET/CT images. The visual analysis was able to diagnose patients and lesions, but was unable to detect differences between imaging performed at different time points (**Table 2**). Therefore, a quantitative analysis was performed to compare the diagnostic efficiency of imaging performed at different time points.

**Table 2:** Comparison of diagnostic performance of 5min and 60min imaging visual analysis in HPT.

		TP	TN	FP	FN	Se	Sp	PPV	NPV	ACC	AUC	*p*
Patients	5min	59	3	4	7	89.39%	42.86%	93.66%	70.00%	86.30%	0.667	0.250
	60min	57	4	3	9	86.36%	57.14%	95.00%	69.23%	82.19%	0.718	
Lesions	5min	94	9	5	30	75.81%	64.29%	94.95%	76.92%	74.64%	0.696	0.063
	60min	90	10	4	34	72.58%	71.43%	95.74%	77.27%	72.46%	0.720	

### 3.3 Quantitative analysis of FCH PET/CT

#### 3.3.1 Comparison of PET parameters between 5- and 60-min images

**Figure 1:**
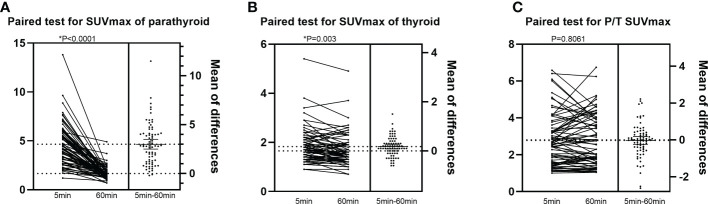
Patient-based analysis of the difference in PET parameters between 5min and 60min imaging.The x-coordinate is the image time, and the y-coordinate are **(A)**: SUVmax of parathyroid, **(B)**: SUVmax of thyroid and **(C)**: P/T SUVmax respectively.

The patient-based analysis showed that there were statistical differences between the 5-min and 60-min P SUVmax (4.65 ± 2.09 vs. 4.23 ± 1.83, *p* < 0.0001)and T SUVmax(1.83 ± 0.72 vs. 1.65 ± 0.67, *p* < 0.003), while no significant difference in P/T between the groups (*p* =0.806, **Figure 1**).

**Figure 2:**
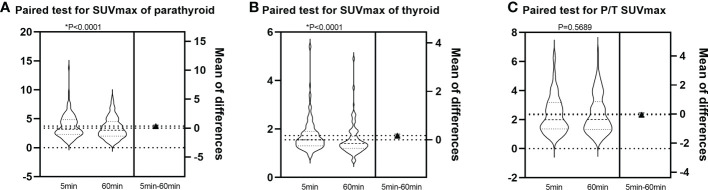
Lesion-based analysis of the difference in PET parameters between 5min and 60min imaging.The x-coordinate is the image time, and the y-coordinate are **(A)**: SUVmax of parathyroid, **(B)**: SUVmax of thyroid and **(C)**: P/T SUVmax respectively.

The lesion-based analysis showed that the P SUVmax at 5 and 60 min was 3.74 ± 1.96 and 3.39 ± 1.78, respectively, whereas the T SUVmax was 1.73 ± 0.69 and 1.55 ± 0.66, respectively (*p* < 0.0001 and *p* < 0.0001, respectively), with no significant difference between the groups (*p* < 0.569; **Figure 2**).

The quantitative analysis showed significant differences in P SUVmax and T SUVmax between the 5- and 60-min images. Conversely, there was no significant difference in P/T SUVmax between the 5- and 60-min images, consistent with the visual analysis, suggesting no significant visual difference on dual-time phase imaging performed at different times.

#### 3.3.2 ROC curve for PET/CT parameters based on patients and lesions

**Figure 3:**
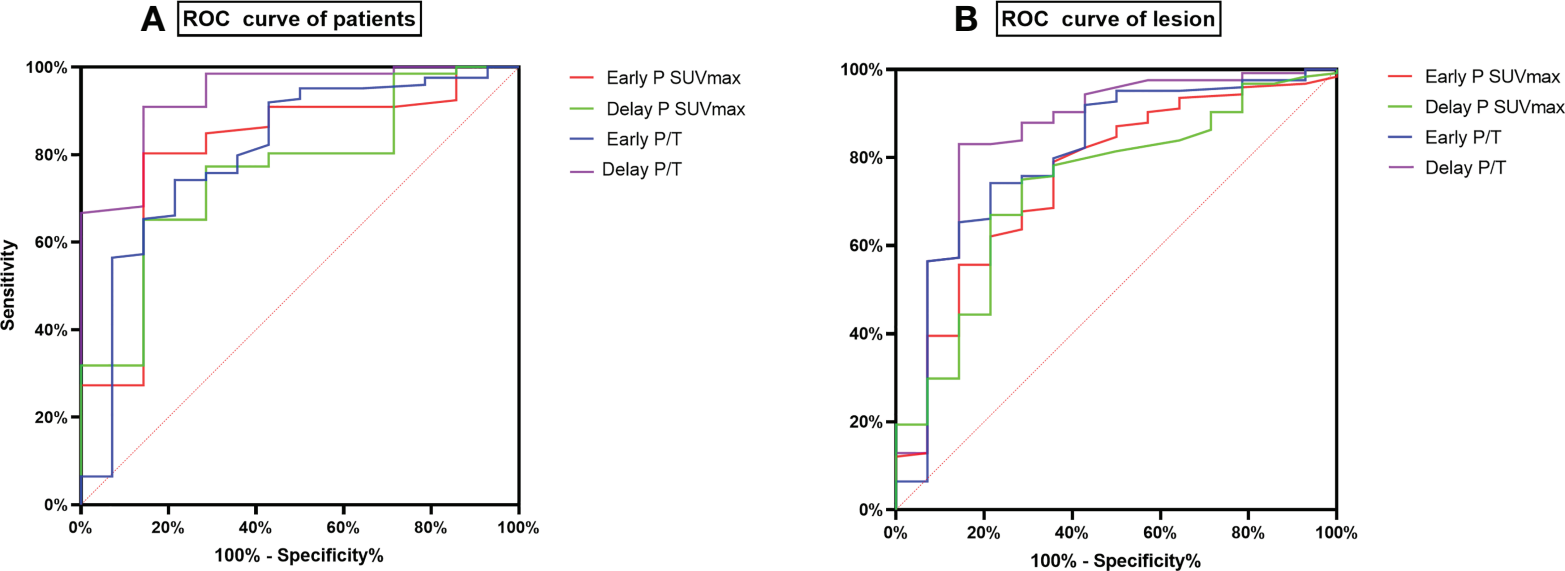
ROC curves of PET parameters based on Patient and lesion. **(A)** is ROC curve of patients, **(B)** is ROC curve of lesions.

The ROC curve analysis of PET/CT quantitative parameters for the diagnosis of HPT patients and lesions showed that imaging performed at 60 min had a high sensitivity and specificity (**Figure 3**). The cutoff P/T SUVmax for the diagnosis of patients was 1.313 (area under the curve [AUC]: 0.934) with a sensitivity of 90.90% and specificity of 85.71%. In comparison, the cut-off P/T SUVmax for the diagnosis of lesions was 1.313 (AUC: 0.855) with a sensitivity of 83.06% and specificity of 85.71% (**Table 3**). The diagnostic accuracy of PET/CT quantitative parameters for the detection of HPT and lesions was higher than that of visual analysis (*p* < 0.05).

**Table 3:** Comparison of the efficacy of PET parameters in patient and lesion detection.

		Se	Sp	PPV	NPV	ACC	AUC	Cut off	*p*
Patients N=73	5min P SUVmax	80.30%	85.71%	98.15%	31.58%	73.97%	0.8095	3.235	0.0074^*^
	60min P SUVmax	65.15%	85.71%	97.72%	20.69%	60.27%	0.7619	3.490	0.0234^*^
	5min P/T	72.73%	100.00%	100.00%	28.00%	65.75%	0.8755	1.955	0.0012^*^
	60min P/T	90.91%	85.71%	98.36%	50.00%	83.56%	0.9340	1.131	0.0002^*^
Lesions N=138	5min P SUVmax	79.03%	64.29%	95.15%	74.28%	74.63%	0.7566	2.350	0.0017^*^
	60min P SUVmax	75.00%	71.43%	95.88%	75.61%	70.29%	0.7353	2.250	0.0040^*^
	5min P/T	74.19%	78.57%	96.84%	74.42%	68.84%	0.8065	1.520	0.0002^*^
	60min P/T	83.06%	85.71%	98.09%	63.64%	76.09%	0.8554	1.310	<0.0001^*^

* P < 0.05, with statisticalsignificance.

In order to rule out the influence of thyroid disease on P/T, our study screened patients with Hashimoto’s thyroiditis by TPO-Ab and ultrasonography, and further analyzed patients without thyroid disease (**Supplementary Table 1**, **Supplementary Figure 1**). The results suggested that 60min P/T had the best diagnostic efficacy, which was consistent with previous results in all patients with thyroid disease included in this study. Therefore, we can conclude that thyroid disease does not have the diagnostic efficacy of imaging P/T for hyperparathyroidism.

### 3.4 PET/CT quantitative parameters for parathyroid adenoma and hyperplasia

**Figure 4:**
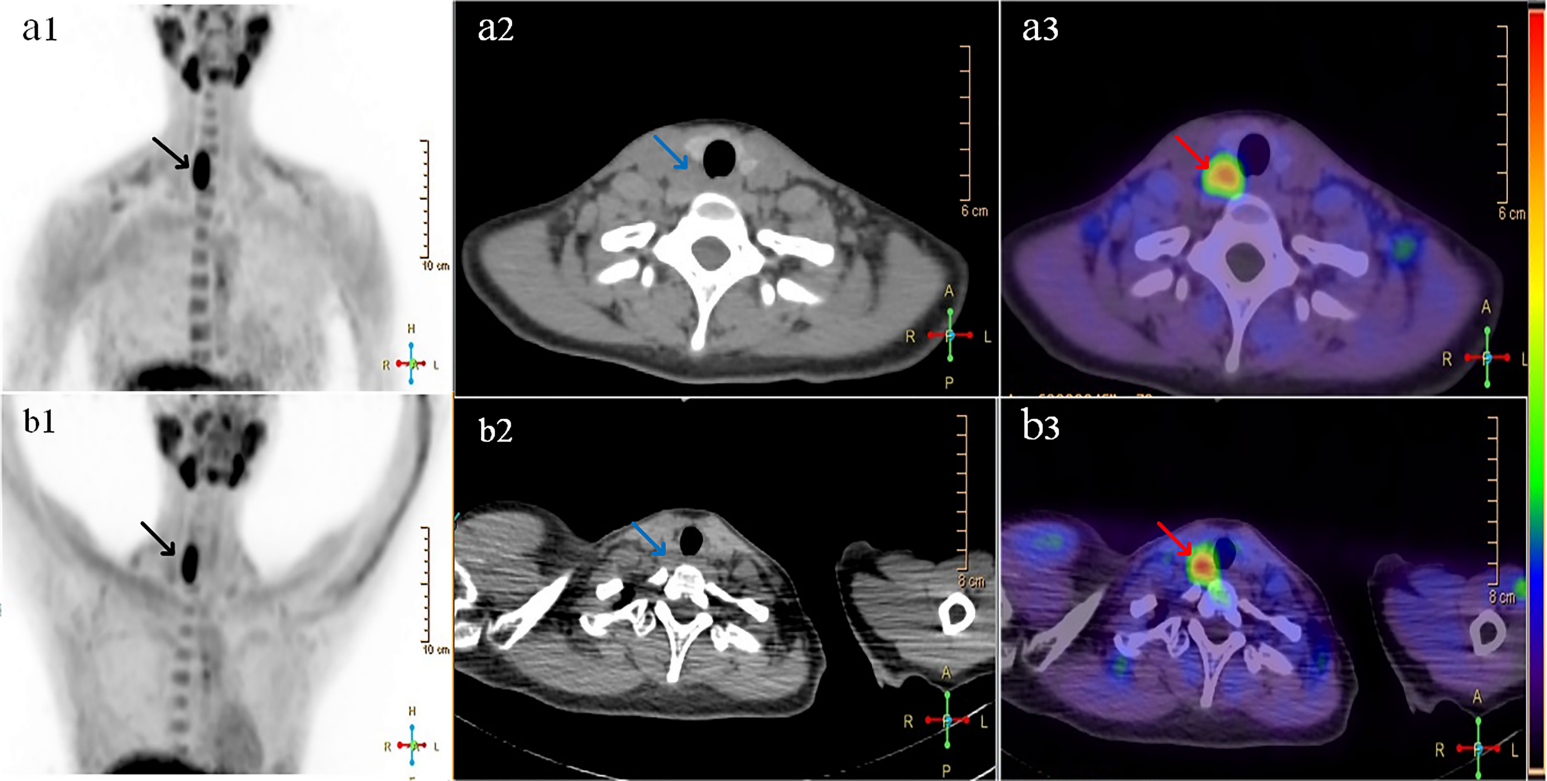
A 28-year-old woman with pain in the lower right back and fatigue of limbs for more than 3 months. The relevant inspection is as follows: PTH 577.3Pg/ml (12-88 Pg/ml), calcium 2.77mmol/L(2.15~2.5mmol/L), 25-hydroxyvitaminD 13.7ng/ml (>20ng/ml). Images named “**(A) **”were 5min ^18^F-FCH imaging: **A1** is 5min PET MIP, black arrow showed ^18^F-FCH radioactive uptake; **A2** was neck CT, blue arrow showed the increased soft tissue density behind the right thyroid; **A3** was PET/CT fusion image, red arrow showed the nodules of ^18^F-FCH metabolic activity, SUVmax was 7.09, SUVmax of adjacent thyroid tissue was 2.20, and P / T was 3.22. Images named “**(B)**” showed 60min 18F-FCH imaging: SUVmax of nodules in **B3** was 8.81, SUVmax of thyroid was 1.90, and P/T was 4.64. Surgical pathology: <lower right parathyroid gland> parathyroid adenoma.

^18^F-FCHPET/CT imaging was able to diagnose HPT patients and lesions. The main pathological causes of HPT were adenoma and hyperplasia. A comparison of PET parameters showed significant differences in the P SUVmax and P/T SUVmax on 5- and 60-min imaging between adenoma and hyperplasia (*p* < 0.05; **Supplementary Table 2**). Furthermore, the P SUVmax on 60-min imaging had the highest diagnostic value at a cutoff value of 3.945 (AUC: 0.783; **Table 4**). **Figure 4** showed ^18^F-FCH PET/CT imaging in a patient with parathyroid adenoma.

**Table 4:** Comparison of diagnostic performance of 5min and 60min imaging of PET parameters in parathyroid adenoma.

	Se	Sp	AUC	Cut off	*p*
5min P SUVmax	60.90%	89.00%	0.777	5.35	<0.0001
5min P/T	73.90%	58.00%	0.663	2.395	0.015
60min P SUVmax	78.30%	75.00%	0.789	3.945	<0.0001
60min P/T	65.20%	67.00%	0.703	2.73	0.003

## 4 Discussion

Several studies have shown that ^18^F-FCH PET/CT is useful for the diagnosis of HPT. Compared to ultrasound and ^99m^Tc-MIBI, ^18^F-FCH PET/CT has a shorter imaging time and higher detection rate. More importantly, it is helpful for accurate location diagnosis of patients with HPT before operation (23, 24). Some previous studies have used dual-time or three-time FCH PET/CT parathyroid imaging for patients with primary parathyroid adenoma. Lezaic and Michaud (2, 25, 26) found that the SUVmax was higher for delayed images than early images and that delayed images had a better target-to-cost ratio. A prospective study by Prabhu et al. (17) suggested that early imaging is sufficient to diagnose lesions. Rep et al. (18) found that early imaging can avoid the missed diagnosis of parathyroid lesions with rapid elution. The aforementioned studies only evaluated the diagnosis of pHPT adenoma patients using FCH PET/CT imaging at different times; however, the results were inconclusive. Additionally, previous studies did not evaluate parathyroid hyperplasia and sHPT simultaneously. Therefore, it is unclear whether ^18^F-FCH PET/CT imaging can distinguish adenoma from hyperplasia.

In the present study, visual and quantitative analyses of images from 73 patients with HPT (pHPT and sHPT) and 138 lesions (including adenomas and hyperplasia) were performed 5 and 60 min after FCH injection for the diagnosis of patients and lesions, respectively. Our results suggest that visual analysis can effectively detect patients and lesions. Quantitative analysis showed that P SUVmax and T SUVmax changed over time, whereas there was no significant difference in P/T SUVmax. The 60-min images had good sensitivity and specificity for the diagnosis of HPT and lesions. Furthermore, the SUVmax of the 60-min images was effective for the diagnosis of parathyroid adenoma and hyperplasia (cutoff: 3.945; AUC: 0.789).

Prabhu et al. (17) prospectively compared 15-min early dynamic imaging with 45-min delayed imaging in patients with pHPT. The results showed that the SUVmax was higher for early imaging and the P/T SUVmax was similar compared to delayed imaging, which is in line with our results. In the present study, the P SUVmax at 5 and 60 min was significantly higher compared to the T SUVmax. Additionally, the SUVmax of the thyroid bed on 60-min images was lower than that on 5-min images, suggesting that the cellular uptake of FCH was significantly increased in HPT, which may be due to increased cell proliferation or pathological changes in blood flow in HPT. Moreover, decreased FCH uptake in HPT and thyroid tissue over time may be due to the metabolic elution of drugs in tissue cells.

Rep et al. (18) found that the sensitivity of imaging was 90.5%, 93.6%, and 93.6% at 5, 60, and 120 min after FCH injection, respectively, whereas the specificity was 98.2% for all and the accuracy was 94.1%, 96.5%, and 96.5%, respectively. Therefore, imaging performed after 60 min of tracer injection had the highest accuracy, which is consistent with our results. In the present study, visual analysis of the diagnosis efficiency of 5- and 60-min images showed a good ability to diagnose patients and lesions (based on patient: *p* = 0.250; based on focus: *p* = 0.063). However, there is no significant difference in the diagnostic value between the 5-min and 60-min imaging of visual analysis. Therefore, we compared the diagnostic efficiency of PET quantitative parameters (P SUVmax, T SUVmax, and P/T SUVmax) on imaging performed at 5 and 60 min based on the patients and lesions, respectively. The ROC curve showed that the P/T SUVmax of 60-min imaging had higher sensitivity and specificity compared to that of 5-min imaging. The best cutoff P/T SUVmax for the diagnosis of patients was 1.313 (AUC: 0.934), which had a sensitivity of 90.90%, specificity of 85.71%, and accuracy of 83.56%. The best cutoff P/T SUVmax for the diagnosis of lesions was 1.313 (AUC: 0.855), which had a sensitivity of 83.06%, specificity of 85.71%, and accuracy of 76.09%. Therefore, the diagnostic efficiency of PET quantitative parameters was significantly higher than that of visual analysis (*p <* 0.05). Quantitative analysis excludes subjective analysis by observers and provides an objective, standardized, and accurate analysis. However, the sensitivity and specificity of quantitative analysis were lower in the present study than in previous studies, possibly because previous studies only included pHPT patients, while our study included pHPT and sHPT patients. It is possible that most sHPT patients have PTH and that certain smaller hyperplastic lesions have less significant FCH uptake (27), which is not prominent on FCH imaging, thereby reducing the overall diagnostic efficiency.

To determine the difference between parathyroid adenoma and hyperplastic lesions on ^18^F-FCH PET imaging, we compared their PET parameters on 5- and 60-min imaging. The SUVmax and P/T SUVmax of adenomas were significantly higher than those of hyperplastic lesions. However, Beheshti et al. (24) found that the SUVmax of adenomas was higher than that of hyperplastic glands, albeit without a significant difference. The difference in ^18^F-FCH PET quantitative parameters between parathyroid adenoma and hyperplasia may be caused by differences in the volume and parenchyma between them. A study (28) has also found that serum calcium, gland size, weight, and largest diameter of adenomas were higher than those of hyperplasia. The parathyroid glands are composed of PTH-secreting master cells, nonfunctional eosinophils, and clear cells, whereas parathyroid adenomas are mostly composed of principal cells. Some studies (29) have found a significantly higher proportion of chief cells and a significantly lower proportion of oxyphil cells in adenomas than in hyperplasia (*p* = 0.0064 and *p* = 0.0054, respectively). Choline is the main component of the cell membrane and a substrate of phosphatidylcholine. Upregulation of phospholipid-dependent choline kinase is associated with PTH overproduction by the parathyroid glands. Therefore, ^18^F-FCH, an analog of choline, may be excessively taken up by functional chief cells, resulting in significantly higher SUVmax and P/T SUVmax values in adenomas than in hyperplasia. The present study and that of Beheshti et al. (24) found that the ^18^F-FCH PET parameters of parathyroid adenoma differed from those of hyperplasia, suggesting that ^18^F-FCH PET is useful for the differentiation of parathyroid adenoma and hyperplasia. Therefore, we further analyzed the diagnostic efficiency of PET parameters in parathyroid adenomas using ROC curve analysis. The results showed that the P SUVmax on 60-min imaging had a higher ability to diagnose parathyroid adenomas and hyperplasia (AUC: 0.789). Lesions with SUVmax > 3.945 were more likely to be diagnosed as parathyroid adenomas.

Studies (30, 31) have found that 20%-40% of patients with hyperparathyroidism are complicated with thyroid autoimmune diseases (mainly Hashimoto’s thyroiditis, HT). To eliminate the effect of thyroid diseases on parathyroid FCH uptake and make the study more rigorous, we carried out **Supplementary data** and analysis. We reviewed the preoperative TPO-Ab levels in 73 patients. Patients with elevated TPO-Ab were considered to be complicated with Hashimoto’s thyroiditis. Among them, 7 patients were complicated with HT, 60 patients had no HT, and 6 patients did not have TPO-Ab. After we re-analyzed 60 patients without HT, found that 60-min P/T had the best diagnostic efficacy, which was consistent with the results of all patients with thyroid disease before this study. In addition, in all included patients’ images, the SUVmax of parathyroid was almost higher than that of thyroid tissue (**Supplementary Table 3**). At the same time, we compared the PET parameters of HPT with HT and without HT and found that there was no significant difference in P SUVmax of 5-min and 60-min between the two groups. The difference in P/T of 5-min and 60-min between the two groups was statistically significant due to the difference in T SUVmax (**Supplementary Table 4**). Although there were few HPT patients complicated with HT in our study, and the sample size was significantly different between the two groups, we can still speculate that thyroid disease will not affect P SUVmax, but may affect P/T. It is worth mentioning that our study is aimed at all people with hyperparathyroidism, there is no restriction on whether to combine other diseases or symptoms. We compared images of the same patient at different time points, not between HPT patients and HPT patients with autoimmune diseases.

Previous studies have mainly focused on the diagnostic rate of FCH imaging in parathyroid adenoma and compared it with ultrasound and ^99m^Tc-MIBI. Our study is the first to differentiate parathyroid adenoma and hyperplasia on ^18^F-FCH PET imaging performed at two time points. Furthermore, we analyzed the ROC curve to detect the optimal cutoff value for the diagnosis of parathyroid adenoma. Our study also had certain limitations. First, only 73 patients were included in this study because of the requirement of postoperative pathological biopsy for comparison. Therefore, we excluded patients who did not undergo surgery and pathological analysis, resulting in a relatively small sample size. Second, previous studies have found that the lesion size may affect FCH uptake; however, due to technical reasons we were unable to measure the lesion size. Additional prospective studies with a large sample size are required to verify our results.

## 5 Conclusion

The performance of dual-time ^18^F-FCH PET imaging for the diagnosis of HPT varies between 5 and 60 min. The 60-min images had higher diagnostic value, whereas the parathyroid SUVmax on 60-min imaging was more effective for the diagnosis of parathyroid adenoma and hyperplasia. Therefore, 60-min imaging may guide the treatment selection for patients with HPT without thyroid diseases.

## Data availability statement

The original contributions presented in the study are included in the article/**Supplementary Material**. Further inquiries can be directed to the corresponding author.

## Ethics statement

The studies involving human participants were reviewed and approved by the ethics committee of the first affiliated Hospital of Chongqing Medical University. Written informed consent for participation was not required for this study in accordance with the national legislation and the institutional requirements. Written informed consent was obtained from the individual(s) for the publication of any potentially identifiable images or data included in this article.

## Author contributions

LX and HP contributed to conception and design of the study. SL and ML organized the database. SL performed the statistical analysis. SL and RZ have been involved in drafting the manuscript. LX and ZW revising it critically for important intellectual content. WL and ZX provided technical support. DW helped with the article revision. All authors contributed to the article and approved the submitted version.
