# Supplementary material for: Clinical application of 18F-FCH PET/CT in the diagnosis and treatment of hyperparathyroidism
Source: Front Endocrinol (Lausanne). 2023 Apr 11;14:1100056. doi: 10.3389/fendo.2023.1100056 (PMC10126393; doi:10.3389/fendo.2023.1100056)
Supplement: Supplementary file 1 [file DataSheet_1.pdf]

Table1 General clinicopathological features of the patients

	Patients (N=73)	pHPT (N=43, 58.90%)	sHPT (N=30, 41.09%)	t/χ^2	*P*
	51.00±14.10	51.02±14.78	50.97±13.37	0.302	0.764
Gender					
M	33 (45.21%)	20	13	0.233	0.630
F	40 (54.79%)	23	17		
Hypercalcemia					
Yes	52 (71.24%)	31	21	0.038	0.846
No	21 (28.76%)	12	9		
Osteoporosis					
Yes	42 (57.53%)	18	24	10.521	0.001*
No	31 (42.47%)	25	6		
Pathology					
Adenoma	21 (61.64%)	18	3	9.028	0.011*
Hyperplastic	45 (28.77%)	21	24		
Others	7 (9.59%)	4	3		
PTH	822.77±955.18	388.33±518.29	1445.46±1093.24	−5.524	0.000*
CT	6.68±18.62	3.45±6.21	11.29±27.70	−1.799	0.076
BAP	50.28±43.14	32.71±31.54	75.45±45.46	−4.747	0.000*
Ca	2.73±0.44	2.85±0.50	2.56±0.27	2.944	0.004*
P	1.18±0.53	0.96±0.30	1.49±0.62	−4.868	0.000*
25-OH VD	17.68±11.27	14.86±5.77	21.87±15.55	−2.7	0.009*
5min P SUVmax	4.65±2.09	4.37±1.92	5.06±2.29	−1.412	0.162
5min T SUVmax	1.83±0.72	1.85±0.62	1.79±0.85	0.335	0.738
5min P/T	2.76±1.41	2.53±1.42	3.09±1.34	−1.685	0.096
60min P SUVmax	4.23±1.83	3.99±1.78	4.56±1.88	−1.306	0.196
60min T SUVmax	1.65±0.76	1.68±0.73	1.60±0.74	0.549	0.585
60min P/T	2.79±1.41	2.56±1.43	3.10±1.34	−1.601	0.114

Table 2 Comparison of diagnostic performance of 5min and 60min imaging visual analysis in HPT

		TP	TN	FP	FN	Se	Sp	PPV	NPV	ACC	AUC	P
Patients	5min	59	3	4	7	89.39%	42.86%	93.66%	70.00%	86.30%	0.667	0.250
	60min	57	4	3	9	86.36%	57.14%	95.00%	69.23%	82.19%	0.718	
Lesions	5min	94	9	5	30	75.81%	64.29%	94.95%	76.92%	74.64%	0.696	0.063
	60min	90	10	4	34	72.58%	71.43%	95.74%	77.27%	72.46%	0.720	

Table 3 Comparison of the efficacy of PET parameters in patient and lesion detection

		Se	Sp	PPV	NPV	ACC	AUC	Cut off	P
Patients N=73	5min P SUVmax	80.30%	85.71%	98.15%	31.58%	73.97%	0.8095	3.235	0.0074*
	60min P SUVmax	65.15%	85.71%	97.72%	20.69%	60.27%	0.7619	3.490	0.0234*
	5min P/T	72.73%	100.00%	100.00%	28.00%	65.75%	0.8755	1.955	0.0012*
	60min P/T	90.91%	85.71%	98.36%	50.00%	83.56%	0.9340	1.131	0.0002*
Lesions N=138	5min P SUVmax	79.03%	64.29%	95.15%	74.28%	74.63%	0.7566	2.350	0.0017*
	60min P SUVmax	75.00%	71.43%	95.88%	75.61%	70.29%	0.7353	2.250	0.0040*
	5min P/T	74.19%	78.57%	96.84%	74.42%	68.84%	0.8065	1.520	0.0002*
	60min P/T	83.06%	85.71%	98.09%	63.64%	76.09%	0.8554	1.310	＜0.0001*

Table 4 Comparison of diagnostic performance of 5min and 60min imaging of PET parameters in parathyroid adenoma

	Se	Sp	AUC	Cut off	P
5min P SUVmax	60.90%	89.00%	0.777	5.35	＜0.0001
5min P/T	73.90%	58.00%	0.663	2.395	0.015
60min P SUVmax	78.30%	75.00%	0.789	3.945	＜0.0001
60min P/T	65.20%	67.00%	0.703	2.73	0.003

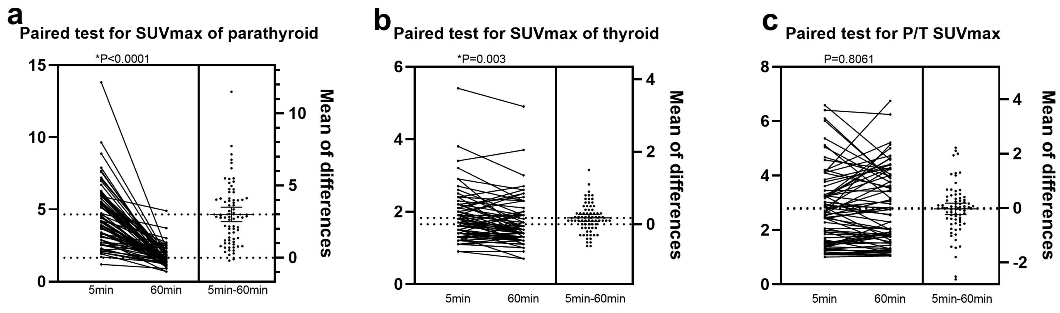

Fig1 Patient-based analysis of the difference in PET parameters between 5min and 60min imaging.The x-coordinate is the image time, and the y-coordinate are (a):SUVmax of parathyroid, (b) :SUVmax of thyroid and (c):P/T SUVmax respectively.

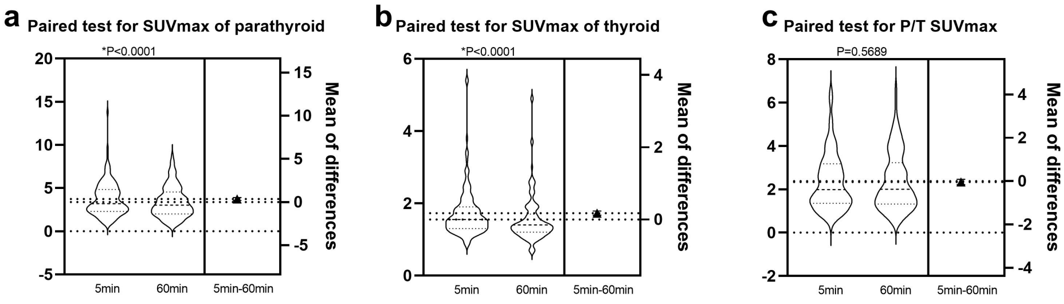

Fig2. Lesion-based analysis of the difference in PET parameters between 5min and 60min imaging.The x-coordinate is the image time, and the y-coordinate are (a):SUVmax of parathyroid, (b) :SUVmax of thyroid and (c):P/T SUVmax respectively.

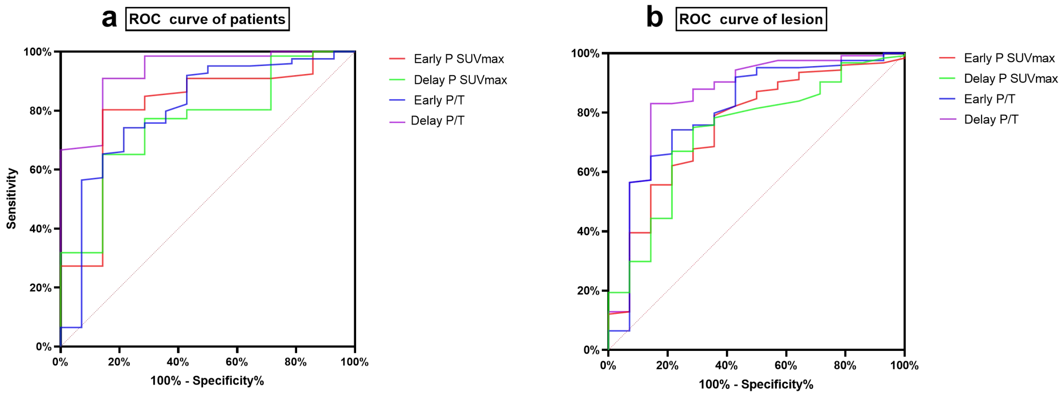

Fig3. ROC curves of PET parameters based on Patient and lesion .(a)is ROC curve of patients, (b)is ROC curve of lesions.

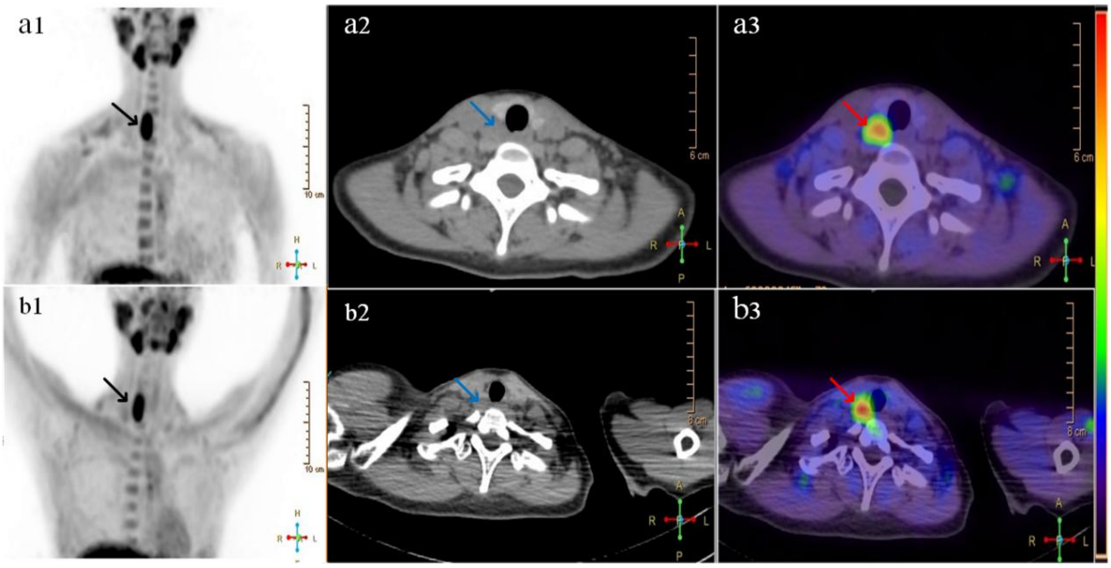

Fig4. A 28-year-old woman with pain in the lower right back and fatigue of limbs for more than 3 months. The relevant inspection is as follows: PTH 577.3Pg/ml (12-88 Pg/ml), calcium 2.77mmol/L(2.15~2.5mmol/L), 25-hydroxyvitaminD 13.7ng/ml (>20ng/ml). Images named “a”were 5min ^{18}F-FCH imaging: a1 is 5min PET MIP, black arrow showed ^{18}F-FCH radioactive uptake; a2 was neck CT, blue arrow showed the increased soft tissue density behind the right thyroid; a3 was PET/CT fusion image, red arrow showed the nodules of ^{18}F-FCH metabolic activity, SUVmax was 7.09, SUVmax of adjacent thyroid tissue was 2.20, and P / T was 3.22. Images named “b” showed 60min 18F-FCH imaging: SUVmax of nodules in b3 was 8.81, SUVmax of thyroid was 1.90, and P/T was 4.64. Surgical pathology: <lower right parathyroid gland> parathyroid adenoma.

Supplementary Table 1 Comparison of the efficacy of PET parameters in patient without thyroid disease

	Sen	Spe	AUC	Cut-off	P
5min P SUVmax	81.48%	100%	0.9228	3.235	0.0007
5min P/T	79.63%	100%	0.8889	1.955	0.0019
60min P SUVmax	72.22%	100%	0.8704	3.490	0.0031
60min P/T	75.93%	100%	0.9475	1.965	0.0004

Supplementary Table 2 Comparison of PET parameters in adenoma and hyperplasia

	Adenoma	Hyperplastic	t	P
5min P SUVmax	5.59±2.52	3.51±1.61	-4.986	＜0.0001
5min T SUVmax	1.88±0.58	1.62±0.49	-2.176	0.032
5min P/T	3.14±1.55	2.29±1.17	-2.937	0.004
60min P SUVmax	5.21±2.04	3.15±1.49	-4.572	＜0.0001
60min T SUVmax	1.7±0.65	1.42±0.40	-2.028	0.053
60min P/T	3.27±1.44	2.34±1.23	-3.217	0.002

Supplementary Table 3 PET parameters in HPT patient with thyroid disease

Patient	Pathology	5min P SUVmax	5min T SUVmax	5min P/T	60min P SUVmax	60min T SUVmax	60min P/T
1	Tissue	5.9	5.4	1.09	5.2	4.9	1.06
2	Hyperplasia	2	1.3	1.54	2.1	1.2	1.75
3	Hyperplasia	4.7	3.4	1.38	4.9	3.7	1.32
4	Hyperplasia	4.2	3.8	1.11	3.2	3	1.07
5	Hyperplasia	3.4	1.4	2.43	2.1	1.4	1.50
6	Hyperplasia	3.41	2.5	1.36	3.28	1.7	1.93
7	Hyperplasia	4.13	1.4	2.95	3.3	1.3	2.54

Supplementary Table 4 Comparison of PET parameters in HPT patient with HT and without HT

	HPT with HT	HPT without HT	t	P
5min P SUVmax	3.963±1.215	4.843±2.198	-1.63	0.131
5min T SUVmax	2.743±1.547	1.73±1.516	3.76	＜0.0001
5min P/T	1.604±0811	2.979±1.432	-3.841	0.003
60min P SUVmax	3.440±1.219	4.429±1.895	-1.895	0.088
60min T SUVmax	2.457±1.438	1.555±0.479	3.574	0.001
60min P/T	1.564±0.570	3.019±1.432	-2.649	0.01

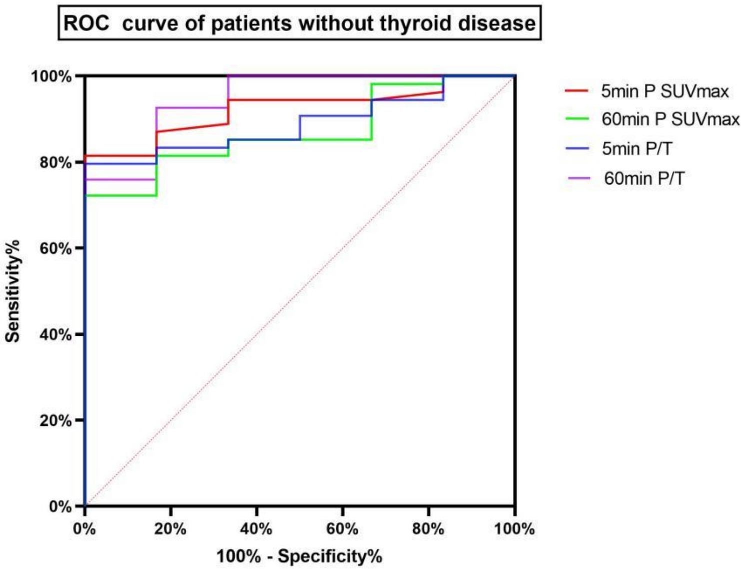

Supplementary Fig1. ROC curves of PET parameters based on Patient without thyroid disease
