# Supplementary figures and images for: Clinical application of 18F-FCH PET/CT in the diagnosis and treatment of hyperparathyroidism
Source: Front Endocrinol (Lausanne). 2023 Apr 11;14:1100056. doi: 10.3389/fendo.2023.1100056 (PMC10126393; doi:10.3389/fendo.2023.1100056)

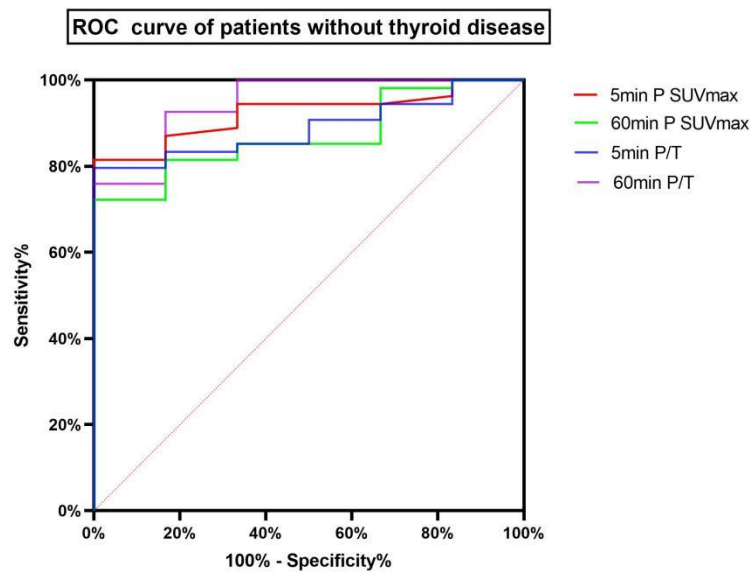

Supplementary Fig1. ROC curves of PET parameters based on Patient without thyroid disease

Supplement: Supplementary file 2 [file Image_1.pdf]
