# Supplementary material for: Clinical application of 18F-FCH PET/CT in the diagnosis and treatment of hyperparathyroidism
Source: Front Endocrinol (Lausanne). 2023 Apr 11;14:1100056. doi: 10.3389/fendo.2023.1100056 (PMC10126393; doi:10.3389/fendo.2023.1100056)
Supplement: Supplementary file 3 [file Table_1.pdf]

Supplementary Table 1 Comparison of the efficacy of PET parameters in patient without thyroid disease

	Sen	Spe	AUC	Cut-off	P
5min P SUVmax	81.48%	100%	0.9228	3.235	0.0007
5min P/T	79.63%	100%	0.8889	1.955	0.0019
60min P SUVmax	72.22%	100%	0.8704	3.490	0.0031
60min P/T	75.93%	100%	0.9475	1.965	0.0004

Supplementary Table 2 Comparison of PET parameters in adenoma and hyperplasia

	Adenoma	Hyperplastic	t	P
5min P SUVmax	5.59±2.52	3.51±1.61	-4.986	<0.0001
5min T SUVmax	1.88±0.58	1.62±0.49	-2.176	0.032
5min P/T	3.14±1.55	2.29±1.17	-2.937	0.004
60min P SUVmax	5.21±2.04	3.15±1.49	-4.572	<0.0001
60min T SUVmax	1.7±0.65	1.42±0.40	-2.028	0.053
60min P/T	3.27±1.44	2.34±1.23	-3.217	0.002

Supplementary Table 3 PET parameters in HPT patient with thyroid disease

Patient	Pathology	5min P SUVmax	5min T SUVmax	5min P/T	60min P SUVmax	60min T SUVmax	60min P/T
1	Tissue	5.9	5.4	1.09	5.2	4.9	1.06
2	Hyperplasia	2	1.3	1.54	2.1	1.2	1.75
3	Hyperplasia	4.7	3.4	1.38	4.9	3.7	1.32
4	Hyperplasia	4.2	3.8	1.11	3.2	3	1.07
5	Hyperplasia	3.4	1.4	2.43	2.1	1.4	1.50
6	Hyperplasia	3.41	2.5	1.36	3.28	1.7	1.93
7	Hyperplasia	4.13	1.4	2.95	3.3	1.3	2.54

Supplementary Table 4 Comparison of PET parameters in HPT patient with HT and without HT

	HPT with HT	HPT without HT	t	P
5min P SUVmax	3.963±1.215	4.843±2.198	-1.63	0.131
5min T SUVmax	2.743±1.547	1.73±1.516	3.76	<0.0001
5min P/T	1.604±0811	2.979±1.432	-3.841	0.003
60min P SUVmax	3.440±1.219	4.429±1.895	-1.895	0.088
60min T SUVmax	2.457±1.438	1.555±0.479	3.574	0.001
60min P/T	1.564±0.570	3.019±1.432	-2.649	0.01
